# Supplementary material for: Efficient probabilistic inference in generic neural networks trained with non-probabilistic feedback
Source: Nat Commun. 2017 Jul 26;8:138. doi: 10.1038/s41467-017-00181-8 (PMC5527101; doi:10.1038/s41467-017-00181-8)
Supplement: Supplementary file 1 — Supplementary Information (PDF) [file 41467_2017_181_MOESM1_ESM.pdf]

File Name: Supplementary Information
Descriptions: Supplementary Figures and Supplementary Table

File Name: Peer Review File
Descriptions:

Supplementary Figures

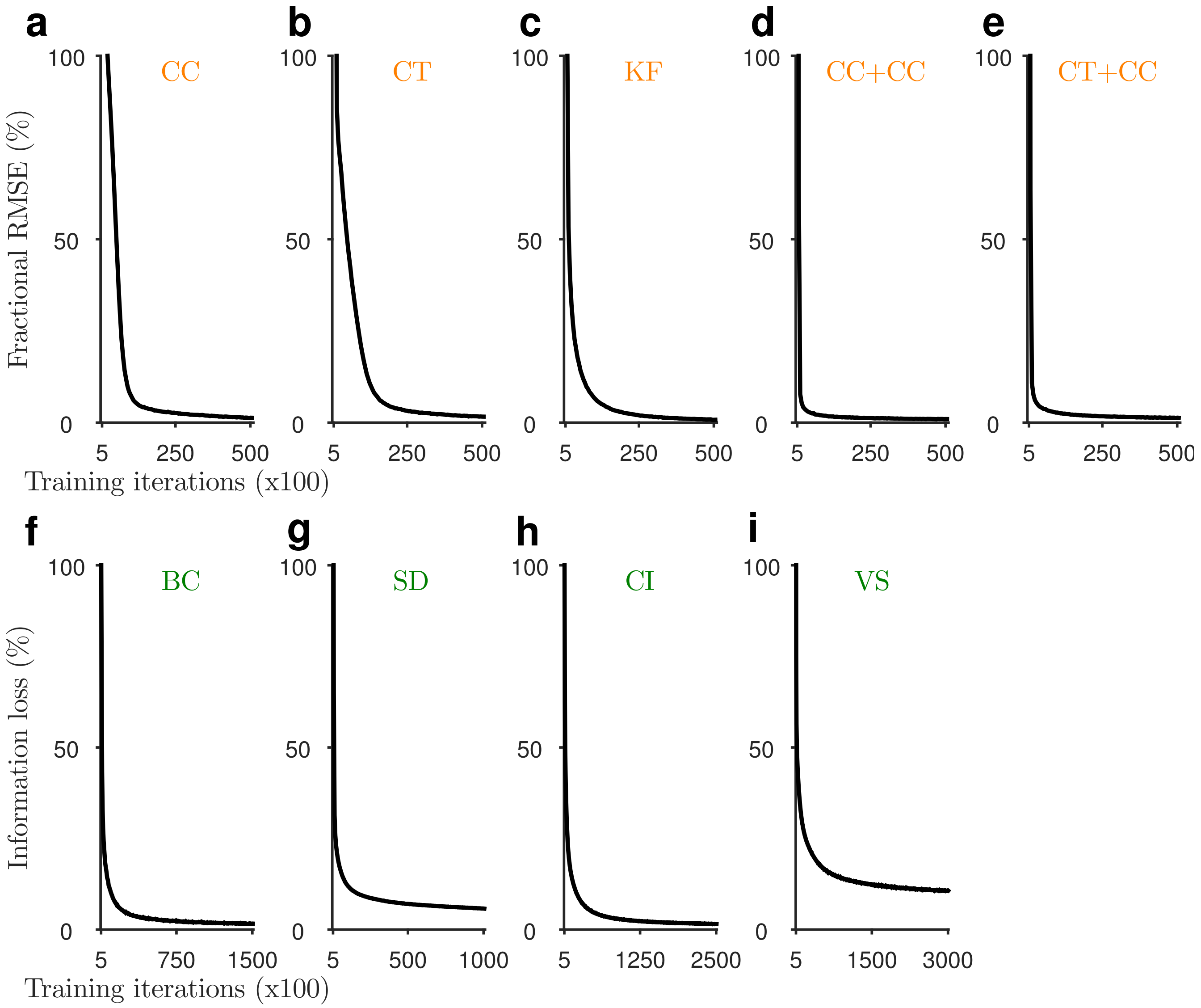

Supplementary Figure 1: Complete learning curves for all tasks in the "all **g**" conditions. Learning curves are averaged over ten independent runs of the simulations.

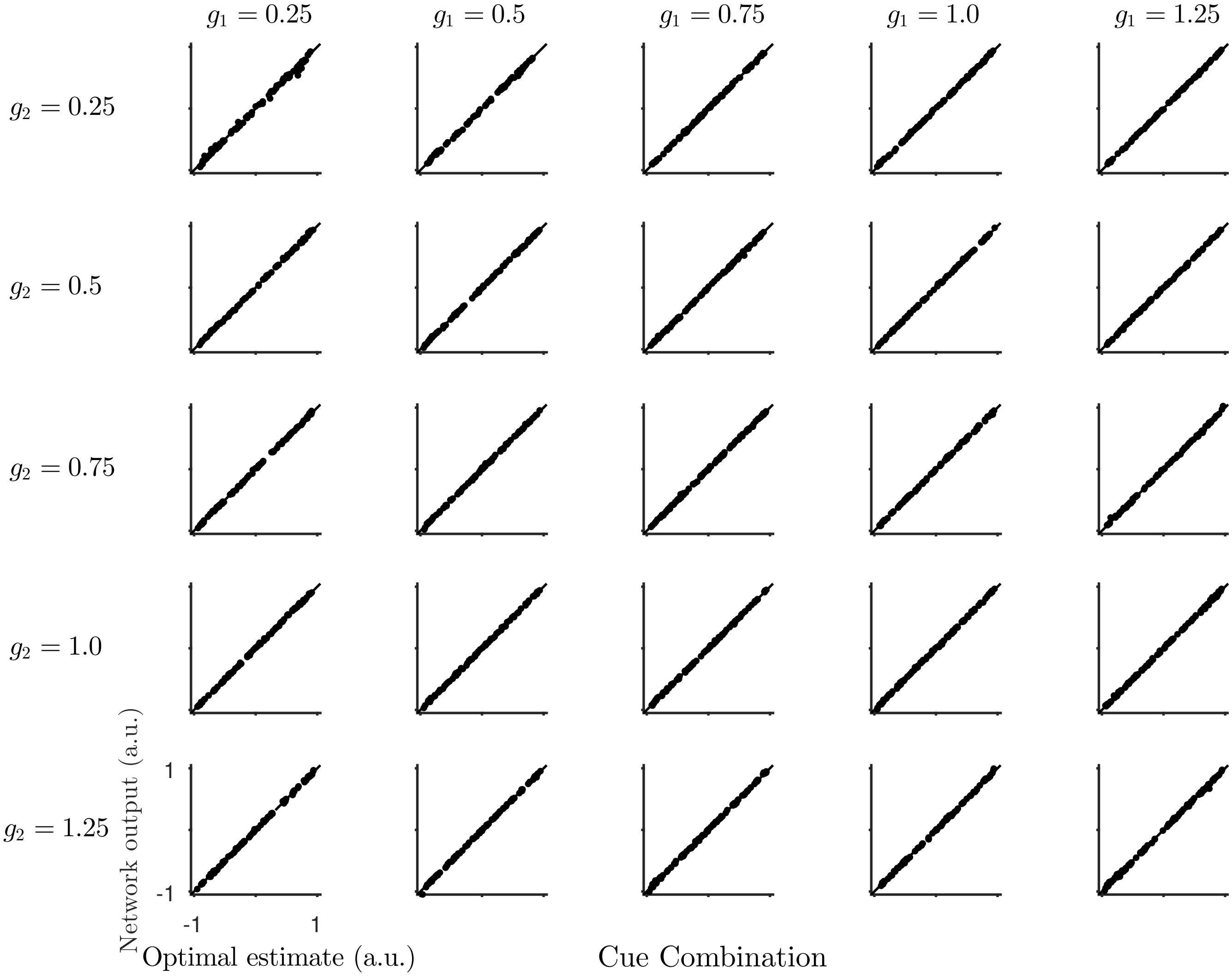

Supplementary Figure 2: Scatterplots of the optimal estimates vs. the outputs of a trained network in "all **g**" condition of the cue combination task. Each subplot corresponds to a different gain combination. The trained network consistently performs well across gain combinations.

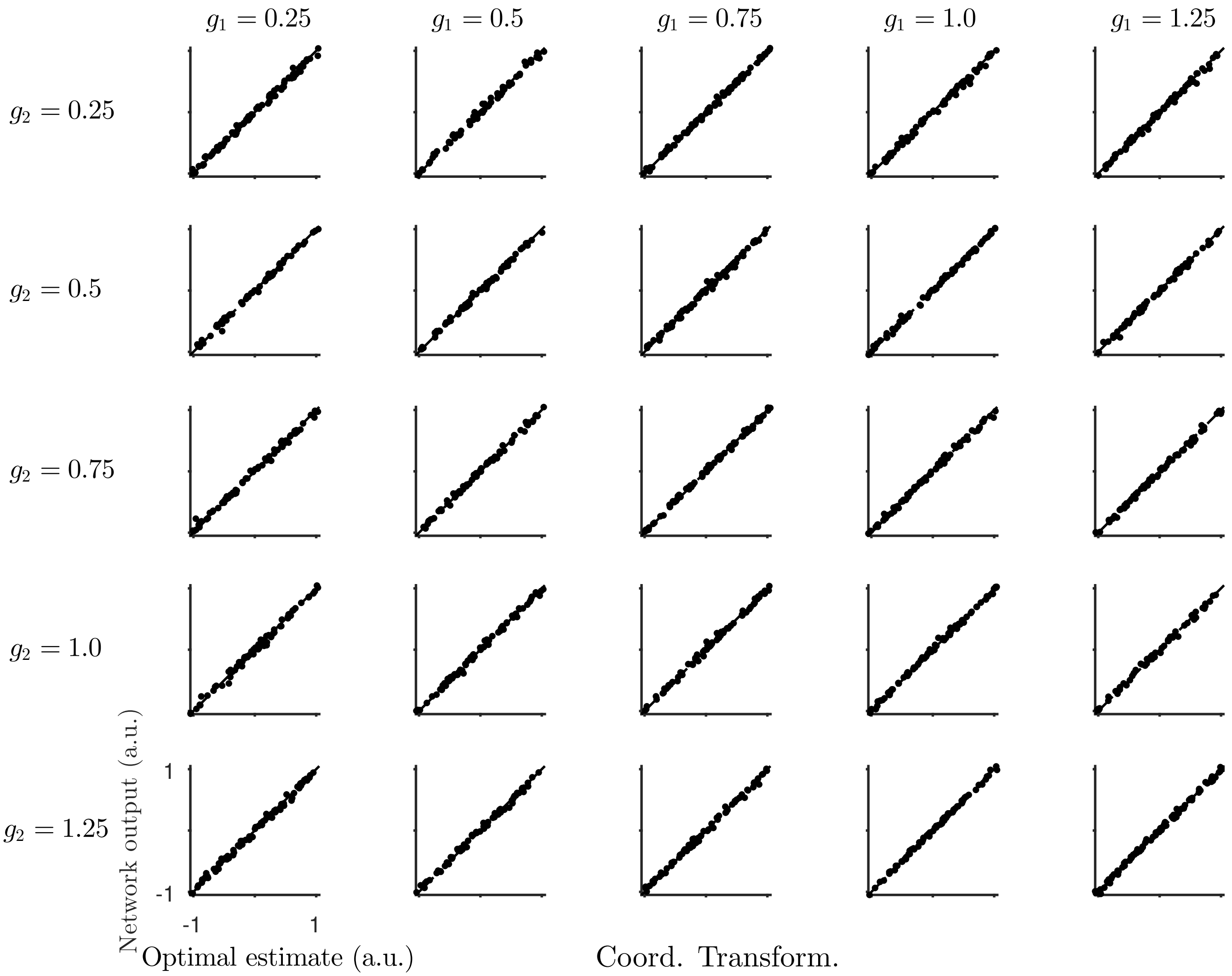

Supplementary Figure 3: Similar to Supplementary Figure 2 but for the coordinate transformation task.

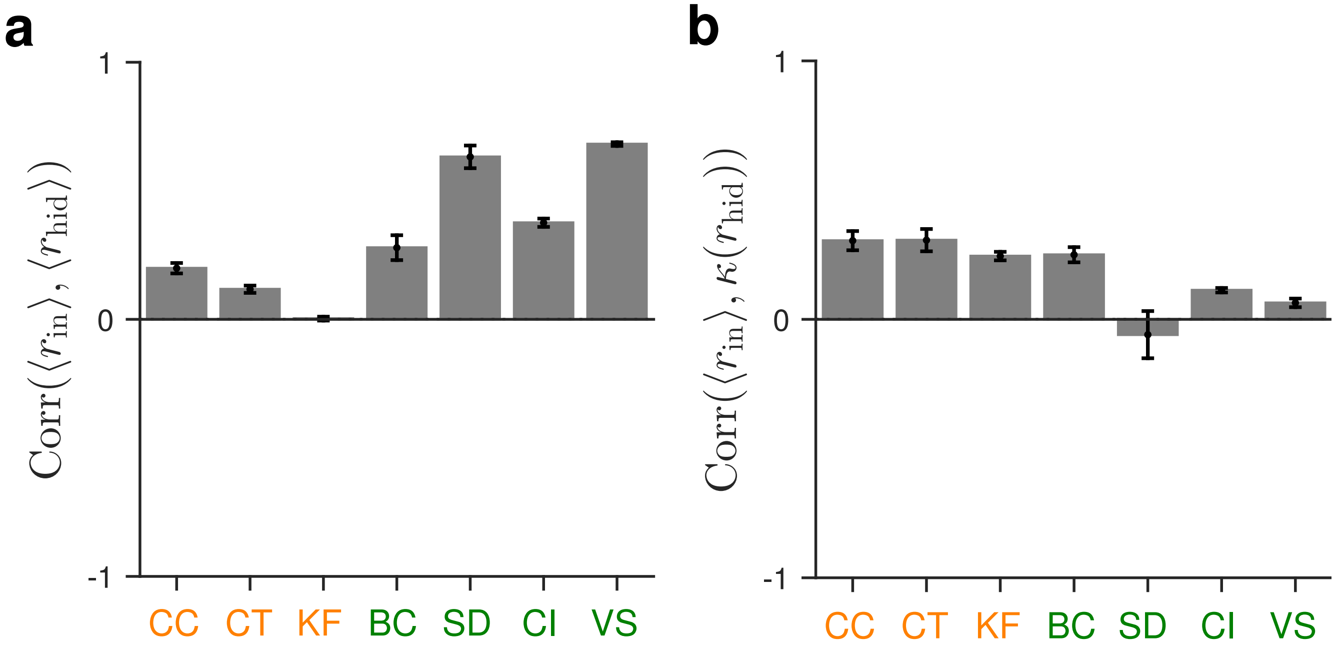

Supplementary Figure 4: Encoding of posterior uncertainty in the recurrent layer of recurrent EI networks. (a) Correlations between mean input and mean hidden (recurrent) layer responses. (b) Correlations between mean input and sparsity (kurtosis) of hidden layer responses. Mean input was calculated by averaging over both input neurons and time points, whereas mean and kurtosis of hidden layer responses were calculated from the hidden layer responses at the final time point. For the Kalman filtering task, mean input was calculated from the input responses at the penultimate time point and the hidden layer statistics were calculated from the hidden layer responses at the final time point. The pattern of correlations is similar to that observed in fully trained feedforward networks (Figure 6a-b).

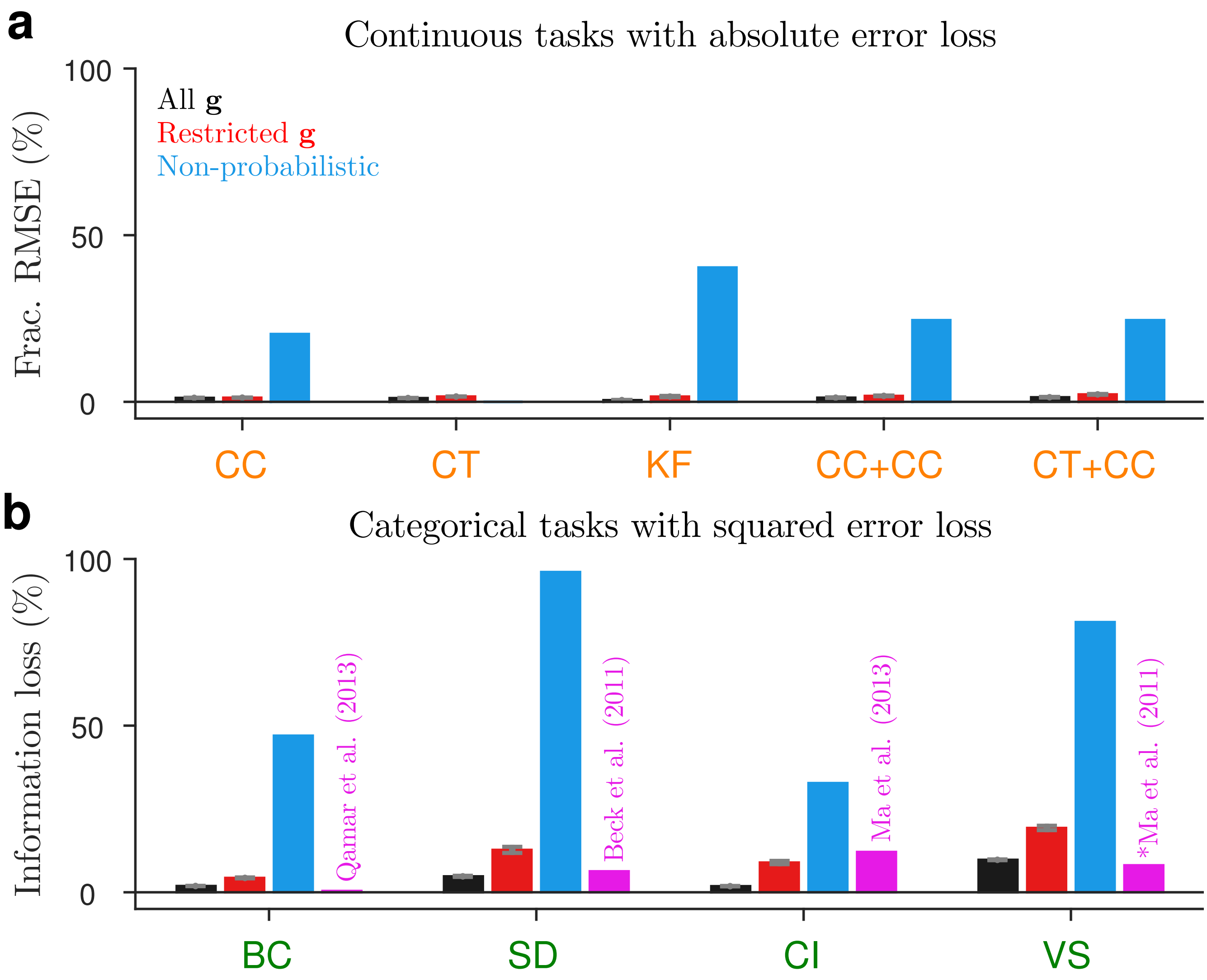

Supplementary Figure 5: Training with other loss functions: (a) Training in continuous tasks with the absolute error loss. Fractional RMSEs were measured with respect to the posterior median estimates. Note that the networks perform well in the modular tasks, demonstrating the implicit encoding of posterior uncertainty as in mean squared error trained networks. (b) Training in categorical tasks with the squared error loss. As in Figure 2d, magenta bars show the performance of hand-crafted networks reported in earlier works. Performance of well-trained feedforward networks are shown here. Error bars represent standard errors over 10 independent runs of the simulations.

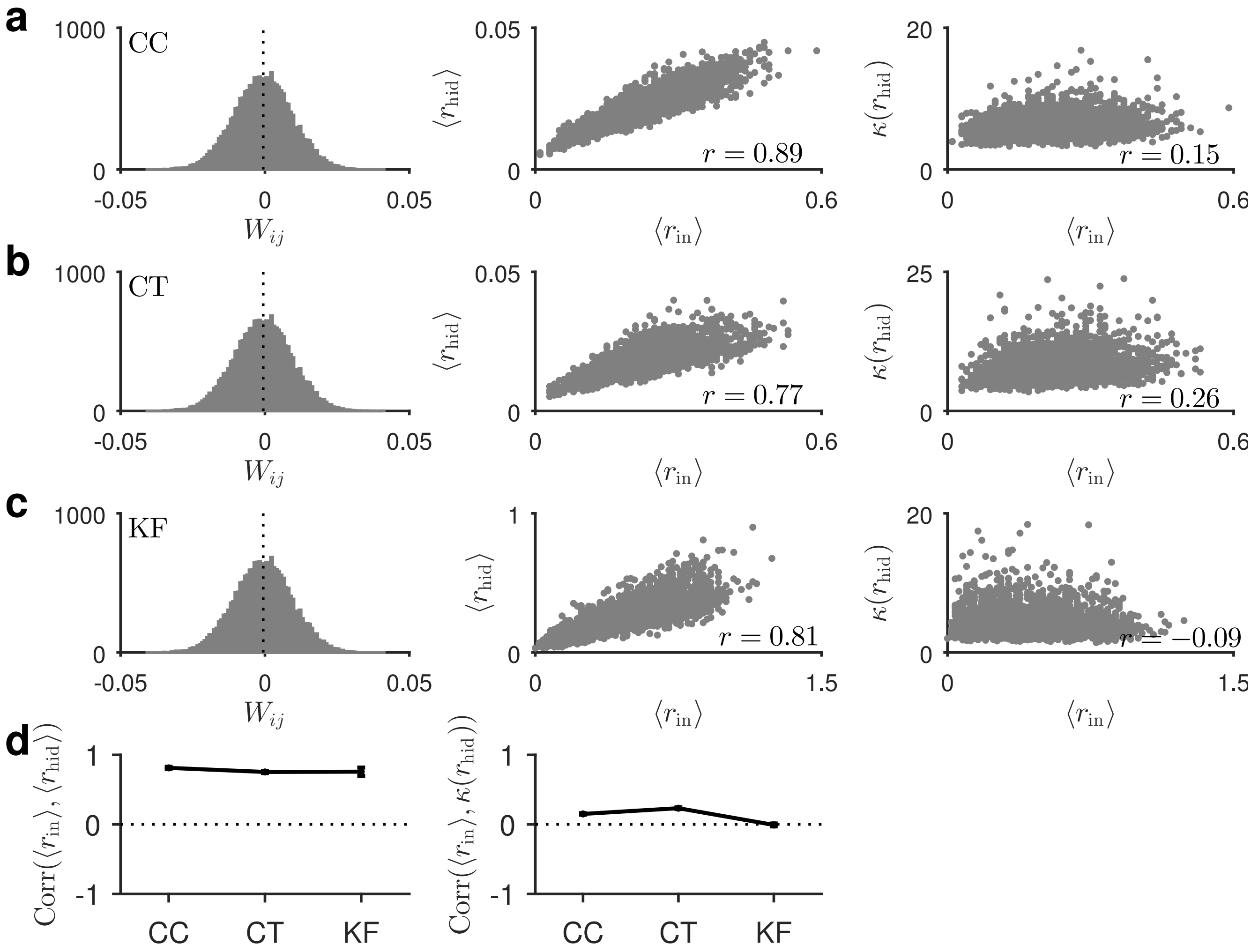

Supplementary Figure 6: Results for the networks with a divisive normalization decoder (generalized center-of-mass decoder) in the continuous tasks: (a) cue combination, (b) coordinate transformation, (c) Kalman filtering. In these networks, the output of the network was given by $\hat{s} = \mathbf{w}^\top \mathbf{r}_{\rm hid} / \mathbf{1}^\top \mathbf{r}_{\rm hid}$. In (a-c), the first column shows the distribution of input-to-hidden layer weights in the trained networks (the distributions are roughly symmetric around zero), the second column is a scatter plot of the mean input vs. the mean hidden layer activity and the third column is a scatter plot of the mean input vs. the kurtosis of hidden layer activity. (d) Correlation between the mean input and the mean hidden unit activity (left) and the correlation between the mean input and the kurtosis of hidden layer activity in the three tasks. Error bars represent standard errors over 10 independent runs of the simulations. In contrast to networks with linear read-outs, networks with a divisive normalization decoder encode posterior precision in the mean hidden layer response, rather than in the sparsity of hidden layer activity, thus approximately preserving the linear decodability of the posterior sufficient statistics.

Supplementary Tables

Task	Slope	95% conf. interval	R^2	p-value
Coord. transformation	0.562	[0.429, 0.695]	0.876	$< 10^{-4}$
Stimulus demixing	0.348	[0.153, 0.543]	0.557	0.002
Cue Combination	0.245	[0.122, 0.368]	0.610	0.001
Kalman filtering	0.210	[0.126, 0.295]	0.711	$< 10^{-3}$
Causal inference	0.176	[0.138, 0.215]	0.892	$< 10^{-4}$
Binary categorization	−0.034	[−0.075, 0.007]	0.215	0.095
Visual search	−0.127	[−0.297, 0.044]	0.268	0.126

Supplementary Table 1: Linear regression statistics for the seven main tasks in Figure 10b ($\log n^*$ vs. $\log d$). The four columns are the estimated slopes, 95% confidence intervals for the slopes, R^2 statistics and p values, respectively. The regressions include an offset term.
